# Deletion of Lytic Transglycosylases Increases Beta-Lactam Resistance in *Shewanella oneidensis*

**DOI:** 10.3389/fmicb.2018.00013

**Published:** 2018-01-22

**Authors:** Jianhua Yin, Yiyang Sun, Yijuan Sun, Zhiliang Yu, Juanping Qiu, Haichun Gao

**Affiliations:** ^1^College of Biotechnology and Bioengineering, Zhejiang University of Technology, Hangzhou, China; ^2^Institute of Microbiology and College of Life Sciences, Zhejiang University, Hangzhou, China; ^3^College of Life Sciences, Nanchang University, Nanchang, China

**Keywords:** lytic transglycosylases, β-lactamase, peptidoglycan, *Shewanella oneidensis*, resistance mechanisms

## Abstract

Production of chromosome-encoded β-lactamases confers resistance to β-lactams in many Gram-negative bacteria. Some inducible β-lactamases, especially the class C β-lactamase AmpC in Enterobacteriaceae, share a common regulatory mechanism, the *ampR*-*ampC* paradigm. Induction of *ampC* is intimately linked to peptidoglycan recycling, and the LysR-type transcriptional regulator AmpR plays a central role in the process. However, our previous studies have demonstrated that the expression of class D β-lactamase gene *blaA* in *Shewanella oneidensis* is distinct from the established paradigm since an AmpR homolog is absent and major peptidoglycan recycling enzymes play opposite roles in β-lactamase expression. Given that lytic transglycosylases (LTs), a class of peptidoglycan hydrolases cleaving the β-1,4 glycosidic linkage in glycan strands of peptidoglycan, can disturb peptidoglycan recycling, and thus may affect induction of *blaA*. In this study, we investigated impacts of such enzymes on susceptibility to β-lactams. Deletion of three LTs (SltY, MltB and MltB2) increased β-lactam resistance, while four other LTs (MltD, MltD2, MltF, and Slt2) seemed dispensable to β-lactam resistance. The double LT mutants Δ*mltB*Δ*mltB2* and Δ*sltY*Δ*mltB2* had β-lactam resistance stronger than any of the single mutants. Deletion of *ampG* (encoding permease AmpG) and *mrcA* (encoding penicillin binding protein 1a, PBP1a) from both double LT mutants further increased the resistance to β-lactams. Notably, all increased β-lactam resistance phenotypes were in accordance with enhanced *blaA* expression. Although significant, the increase in β-lactamase activity after inactivating LTs is much lower than that produced by PBP1a inactivation. Our data implicate that LTs play important roles in *blaA* expression in *S. oneidensis*.

## Introduction

Beta-lactam antibiotics are the most widely used group of antibiotics, they target the penicillin-binding proteins (PBPs), eventually disrupting the peptidoglycan synthesis. To combat these antibiotics, microorganisms have evolved multiple resistance mechanisms, including the direct inactivation or modification of antibiotics, protection of antibiotic targets, overexpression of drug efflux pumps, and reduction of permeability of the outer membrane (Blair et al., 2015). In Gram-negative bacteria, the production of β-lactamases is the predominant strategy of resistance to β-lactams. These enzymes, which resemble PBPs structurally and mechanistically, have ability to rapidly hydrolyze the β-lactams (Nicholas and Davies, 2012).

A great number of chromosome- and plasmid-mediated β-lactamases have been characterized. Expression of β-lactamases encoded by the chromosome is often inducible by β-lactam antibiotics. The regulatory mechanisms for β-lactamase production are intensively studied, especially for class C β-lactamase AmpC in several members of family *Enterobacteriaceae* (such as *Enterobacter cloacae, Citrobacter freundii*) (Lindberg et al., 1985; Jacobs et al., 1994, 1997) and *Pseudomonas aeruginosa* (Juan et al., 2006, 2017; Moya et al., 2009; Ropy et al., 2015). The induction of *ampC* is intimately linked to peptidoglycan recycling through LysR-type transcriptional regulator AmpR (the *ampC*-*ampR* paradigm). The peptidoglycan fragments, usually GlcNAc-1,6-anhMurNAc peptides, enter the cytoplasm across the inner membrane via permease AmpG. In the cytoplasm, these fragments are further processed by NagZ and AmpD, and then participate in peptidoglycan biosynthesis again. Two cytoplasmic peptidoglycan intermediates can act as regulatory ligands for *ampC* induction by binding to AmpR, resulting in either activation or repression of *ampC* expression.

In contrast to the *ampC*-*ampR* paradigm, our previous studies demonstrated that the regulatory mechanism for β-lactamase induction is distinct in *Shewanella oneidensis* MR-1, the most intensively studied strain in *Shewanella* genus (Yin et al., 2014). This genus belongs to the order “Alteromonadales” within the class “γ-proteobacteria”. *Shewanella* species are widely distributed in marine and freshwater environments and well-known for their diverse metabolic capabilities and versatile electron-accepting capacities (Fredrickson et al., 2008). Recently, *Shewanella* species are increasingly being implicated as human pathogens (Janda and Abbott, 2012; Cimmino et al., 2016). More importantly, bacteria of the genus *Shewanella* are regarded as a reservoir for antibiotic resistance, since several β-lactamases and Qnr-type quinolone resistance determinants have been isolated and characterized from this genus (Poirel et al., 2004). In *S. oneidensis* MR-1, the production of class D β-lactamase BlaA (encoded by the *blaA* gene) confers resistance to penicillins and the expression of *blaA* is induced by ampicillin (AMP) (Yin et al., 2013). However, a homolog of AmpR is absent in *S. oneidensis* and major peptidoglycan recycling enzymes (AmpG, NagZ and AmpD) have opposite effects for β-lactamase expression when compared to the *ampC*-*ampR* paradigm (Yin et al., 2014). Inactivation of PBP1a and its lipoprotein cofactor (LpoA) results in constitutive expression of *blaA* (Yin et al., 2015). Therefore, *S. oneidensis* contains an AmpG-independent, but PBP1a-dependent inducible pathway for *blaA* expression.

A common feature for the induction of β-lactamase genes is the involvement of peptidoglycan recycling. In contrast to the peptidoglycan recycling event in the cytoplasm, how the precursors of regulatory ligands are produced in the periplasm remains unclear. At least three classes of enzymes are responsible for peptidoglycan hydrolysis, including low-molecular-weight PBPs (LMW PBPs), N-acetylmuramoyl-L-alanine amidases and lytic transglycosylases (LTs) (van Heijenoort, 2011). Among them, LMW PBPs possess endopeptidase and/or carboxypeptidase activities, responsible for controlling the extent of cross-linking. In *P. aeruginosa*, inactivation of LMW PBPs increased *ampC* expression and β-lactam resistance (Moya et al., 2009; Ropy et al., 2015). N-acetylmuramoyl-L-alanine amidases liberate the peptides from the carboxyl of the lactyl moiety of MurNAc. LTs cleave the β-1,4 glycosidic linkage between GlcNAc and MurNAc resides, leading to the formation of GlcNAc-1,6-anhMurNAc peptides. The regulatory ligands for *ampC* induction are very likely derived from the function of LTs (Kraft et al., 1999). However, Gram-negative bacteria harbor multiple LTs that may be functionally redundant. For example, *Escherichia coli* encodes eight LTs (Yunck et al., 2016), while *P. aeruginosa* possesses 11 LTs (Lee et al., 2017). Based on the sequence similarities and identified consensus motifs, LTs are classified into families 1–4 (Blackburn and Clarke, 2001); two forms of LTs exist in bacteria, including soluble (sLTs) and membrane-bound (mLTs) LTs. Recent studies demonstrated that loss of SltB1 or MltB increases β-lactam resistance, whereas loss of Slt decreases resistance in *P. aeruginosa* (Cavallari et al., 2013; Lamers et al., 2015). In *Stenotrophomonas maltophilia*, inactivation of *mltD1* confers a partial basal-level β-lactamase derepression phenotype (Huang et al., 2015). Besides, very little research has been focused on LTs and β-lactam resistance in other bacteria. The effect of LTs on *blaA* expression in *S. oneidensis* remains unknown.

In this study, our goal is to explore the roles of LTs in β-lactam resistance in *S. oneidensis* MR-1. *S. oneidensis* possesses seven genes that are predicted to encode LTs. Inactivation of three LTs [MltB(SO1166), SO1194, and SltY(SO2040)] increased the expression of *blaA*, resulted in improved resistance to β-lactams. Further studies suggested that these three LTs have additive effects for the expression of *blaA* and β-lactam resistance. Our results implicated that these LTs are involved in *blaA* expression and β-lactam resistance in *S. oneidensis*.

## Materials and methods

### Bacterial strains, plasmids, and culture conditions

All bacterial strains and plasmids used in this study are listed in Table 1. All primers were synthesized by Sangon Biotech (Shanghai, China) and listed in Table S1. For normal growth, *S. oneidensis* and *E. coli* were cultivated aerobically in Luria-Bertani (LB) medium (Difco, Detroit, MI) at 30°C and 37°C, respectively. Where needed, the growth medium was supplemented with chemicals at the following concentrations: 2,6-diaminopimelic acid (DAP), 0.3 mM; ampicillin (AMP), 100 μg/mL; kanamycin (Km), 50 μg/mL; and gentamicin (Gm), 15 μg/mL. All chemicals were purchased from Sigma-Aldrich (Shanghai, China) unless otherwise noted.

**Table 1:** Bacterial strains and plasmid used in this study.

**Strain or plasmid**	**Description**	**Source or reference**
***E. COLI*** **STRAINS**		
DH5α	Host strain for plasmids	Lab stock
WM3064	Donor strain for conjugation; Δ*dapA*	W. Metcalf, UIUC^a^
***S. ONEIDENSIS*** **STRAINS**		
MR-1	Wild-type	ATCC 700550
HG0280	Δ*mrcA* derived from MR-1	Yin et al., 2015
HG0837	Δ*blaA* derived from MR-1	Yin et al., 2013
HG1166	Δ*mltB* derived from MR-1	This study
HG1994	Δ*mltB2* derived from MR-1	This study
HG2040	Δ*sltY* derived from MR-1	This study
HG2564	Δ*mltD2* derived from MR-1	This study
HG3288	Δ*mltF* derived from MR-1	This study
HG3814	Δ*ampG* derived from MR-1	Yin et al., 2014
HG4017	Δ*mltD* derived from MR-1	This study
HG4660	Δ*slt2* derived from MR-1	This study
HG1166-0837	Δ*mltB*Δ*blaA* derived from MR-1	This study
HG1994-0837	Δ*mltB2*Δ*blaA* derived from MR-1	This study
HG2040-0837	Δ*sltY*Δ*blaA* derived from MR-1	This study
HG1166-1994	Δ*mltB*Δ*mltB2* derived from MR-1	This study
HG1994-2040	Δ*sltY*Δ*mltB2* derived from MR-1	This study
HG1166-1994-0280	Δ*mltB*Δ*mltB2*Δ*mrcA* derived from MR-1	This study
HG1994-2040-0280	Δ*sltY*Δ*mltB2*Δ*mrcA* derived from MR-1	This study
HG1166-1994-3814	Δ*mltB*Δ*mltB2*Δ*ampG* derived from MR-1	This study
HG1994-2040-3814	Δ*sltY*Δ*mltB2*Δ*ampG* derived from MR-1	This study
**PLSMIDS**		
pHGM01	Ap^r^ Gm^r^ Cm^r^; suicide vector	Jin et al., 2013
pHGEI01	Integrative *lacZ* reporter vector	Fu et al., 2014
pHG101	Promoterless vector for complementation	Wu et al., 2011
pHG101-*sltY*	pHG101 containing the *sltY* and its promoter	This study
pHG101-*mltB*	pHG101 containing the *mltB* and its promoter	This study
pHG101-*mltB2*	pHG101 containing the *mltB2* and its promoter	This study
pHG101-*mltF*	pHG101 containing the *mltF* and its promoter	This study

a *UIUC, University of Illinois at Urbana-Champaign*.

### In-frame deletion mutagenesis and complementation

In-frame deletion strains of *S. oneidensis* MR-1 were constructed by the *att*-based fusion PCR method (Jin et al., 2013). In brief, two fragments flanking the targeted gene were amplified by PCR with primers containing *attB* sequence or gene-specific sequence, and then joined together by an overlapping PCR method. The fusion fragments were introduced into plasmid pHGM01 by site-specific recombination, using BP Clonase (Invitrogen), and then transformed into *E. coli* WM3064 strain (DAP auxotroph). The resulting recombinant plasmids were transferred from *E. coli* WM3064 into the appropriate *S. oneidensis* strains via conjugation. Integration of the recombinant plasmid into the chromosome was selected by resistance to gentamicin and verified by PCR. The correct transconjugants were grown in LB broth in the absence of NaCl and plated onto LB medium supplemented with 10% sucrose. Gentamicin-sensitive and sucrose-resistant colonies were screened by PCR for deletion of the targeted gene. The deletion mutations were then verified by sequencing.

Plasmid pHG101 was used for genetic complementation of mutants (Wu et al., 2011). A fragment containing the gene of interest together with its native promoter was amplified by PCR and cloned into pHG101. The correct recombinant plasmids were transferred into its corresponding mutant strains via conjugation. The presence of recombinant plasmid was confirmed by plasmid purification and restriction enzyme digestion.

### Growth of *S. oneidensis*

For measuring growth of different *S. oneidensis* strains, Overnight cultures were diluted 1:100 in 3 mL fresh LB medium and incubated at 200 rpm 30°C. The optical density at 600 nm (OD600) was recorded every 1 h. Then the generation times (G) were calculated according to the OD600 at the exponential stage.

### Antibiotic susceptibility assay

Antibiotic susceptibility of *S. oneidensis* was determined with both liquid and solid cultures. Liquid cultures were utilized to determine the minimum inhibitory concentration (MIC) of the antibiotics (Yin et al., 2014). Antibiotics used in the MICs assay were AMP, cefotaxime (CTX), and imipenem (IPM). All MICs were determined at least in triplicate. Susceptibility assays on plates were also used to compare differences in AMP resistance among *S. oneidensis* strains. In this case, decimal dilution series were prepared. Three μL of each dilution was placed onto LB plates supplemented with AMP at different concentrations. The plates were incubated for 18 h at 30°C and then photographed.

### Promoter activity assay

The activity of the promoter for each LT and *blaA* genes was determined by a markerless integrative *lacZ* reporter system, which we used to measure the activity of the *blaA* promoter (Yin et al., 2014, 2015). In brief, the wild type strains harboring different transcriptional fusion vector were cultivated to the exponential-phase ([OD600] of ≈ 0.4), then harvested by centrifugation at 4°C. After washed with phosphate-buffered saline (PBS) and treated with lysis buffer [0.25 M Tris-HCl (pH = 7.5), 0.5% Triton-X-100]. β-Galactosidase activity was determined by monitoring optical density at 420 nm using a Infinite M200 Pro microplate reader (Tecan) as previously described (Yin et al., 2014, 2015).

### BlaA β-lactamase activity assay

The β-lactamase activity was measured directly by nitrocefin hydrolysis method, as described previously (Yin et al., 2014). In brief, bacterial cultures were cultivated to the early-exponential-phase [(OD600) of ≈ 0.2], and then 0 or 200 μg/mL AMP was added for *blaA* induction. The cultures were incubated for an additional 2 h at 30°C and 200 rpm. After incubation, 1 mL culture was centrifuged at 2,500 × g for 5 min, washed once with 1 mL 50 mM phosphate buffer (pH 7.0), and resuspended in 1 mL of the same buffer. Crude cell extracts were prepared by sonication (pulse on, 3 s; pulse off, 4 s; 10 times). The protein content of the crude extracts was determined using a Bradford assay with BSA as a standard (Bio-Rad). The nitrocefin hydrolysis assays were performed in 200 μL reaction mixtures containing 8 μg total protein and 4 μg nitrocefin (Calbiochem, San Diego, CA). Nitrocefin hydrolysis was measured every minute for 10 min at 25°C by absorbance at 486 nm. The specific BlaA β-lactamase activity was expressed in nanomoles of nitrocefin hydrolyzed per minute per milligram of protein. All experiments were performed in triplicate, and the results presented are averages for the three experiments.

### Other analyses

The experimental values were subjected to statistical analyses and are presented as means ± standard deviations. Student's *t*-test was performed for pairwise comparisons of groups.

## Results

### LTs in *S. oneidensis* MR-1

According to genome annotation, seven genes [*mltB* (SO1166), *SO1994, sltY* (SO2040), *SO2564, mltF* (SO3288), *mltD* (SO4017), and *SO4660*] are predicted to encode LTs in *S. oneidensis* MR-1. Except for SO4660, all LTs have a homolog in *E. coli* and *P. aeruginosa* (Table 2). SltY and MltF share 29 and 41% sequence identity with *E. coli* Slt70 and MltF, respectively. Interestingly, both MltB and SO1994 are *E. coli* MltB homologs (36 and 35% identity respectively); both SO2564 and MltD are homologous with *E. coli* MltD (34 and 33% identity respectively). These results indicate that *S. oneidensis* may produce two MltB and MltD isozymes. SO4660 is a protein of 239 amino acids (aa), which contains a transglycosylase SLT domain at the C-terminal region (115–217 aa). Blastp results demonstrate that SO4660 shares 37% identity and 51% similarity with *P. aeruginosa* Slt only at the C-terminal region (Query cover: 52%; *E*-value: 6e-17). The amino acids of SltY and SO4660 do not include a predicted lipoprotein process site, suggesting that they are soluble LTs. However, the rest LTs contain a predicted lipidation site [L(A/S)(G/A)C], a hallmark for lipoprotein (Tokuda and Matsuyama, 2004), implicating that these enzymes are membrane-bound lipoprotein. *E. coli* Slt70, MltF, MltB, MltD, and *P. aeruginosa* Slt belong to family 1A, 1E, 3, 1D, and 1A, respectively (Blackburn and Clarke, 2001). The consensus motifs of each family are also found in *S. oneidensis* counterparts (Figure S1). Therefore, based on sequence similarities and consensus motifs, we designated *SO1994, SO2564*, and *SO4660* of *S. oneidensis* as *mltB2, mltD2*, and *slt2*, respectively. In total, *S. oneidensis* produces seven LTs, two from families 1A (SltY, Slt2), 1D (MltD and MltD2), and 3 (MltB and MltB2), one from family 1E (MltF).

**Table 2:** Lytic transglycosylases in *S. oneidensis*.

**Locus**	**Gene**	***E. coli* or *P. aeruginosa* counterparts^a^**	**Similarity (%)**	**Identity (%)**	**Query Cover (%)**	***E*-Value**	**Family**
SO1166	*mltB*	MltB	50	36	96	5e-60	3
SO1994	*mltB2*	MltB	50	35	66	6e-33	3
SO2040	*sltY*	Slt70	50	29	95	5e-90	1A
SO2564	*mltD2*	MltD	56	34	95	1e-83	1D
SO3288	*mltF*	MltF	60	41	92	5e-120	1E
SO4017	*mltD*	MltD	52	33	96	1e-79	1D
SO4660^b^	*slt2*	Slt	51	37	52	6e-17	1A

a *SO4660 is compared to P. aeruginosa counterpart, whereas other LTs are compared to E. coli counterparts*.

b *SO4660 and P. aeruginosa Slt share only partially identity at the C-terminal region*.

**Figure 1:**
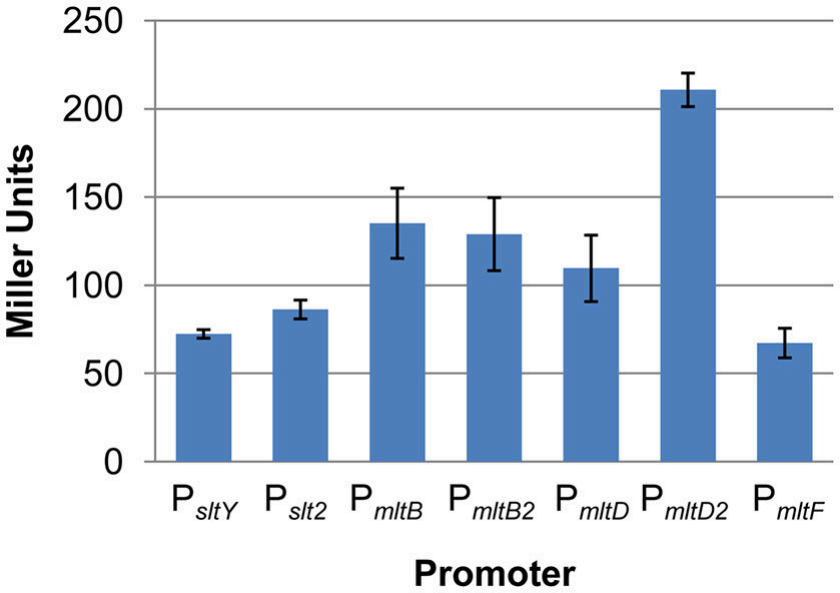
All LTs are expressed in *S. oneidensis*. Promoter activity of each LT gene was determined by measuring β-galactosidase (in Miller Units) using the markerless integrative *lacZ* reporter system in the wild type strain. Results are averages for at least three replicates, and the error bars represent standard derivation (SD).

To determine whether all these LTs are expressed, a markerless integrative *lacZ*-reporter system was employed to measure the promoter activity of each LT genes (Fu et al., 2014; Figure 1). Compared to the control, the expression of the β-galactosidase driven by each promoter was detectable in the wild type background. Among these, the promoter of *mltD2* (P_*mltD*2_) had the highest activity to induce the expression of *lacZ*, while the promoter of *mltF* (P_*mltF*_) had the lowest activity. These results suggest that all LT genes are effectively expressed in our tested conditions.

### Effects of LTs on cell growth

**Figure 2:**
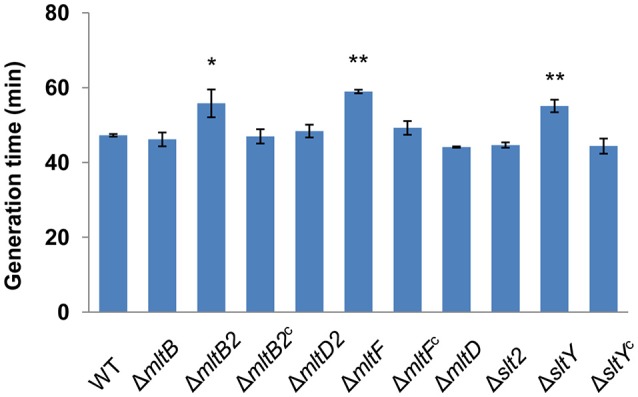
Effects of LTs on cell growth. The generation times for different strains were calculated from the OD600 values. Δ*mltB2*^c^, Δ*mltF*^c^ and Δ*sltY*^c^ represent Δ*mltB2*, Δ*mltF* and Δ*sltY* that were complemented with pHG101 *in trans*, respectively. Results are averages for at least three replicates, and the error bars represent standard derivation (SD) (^*^P < 0.05 and ^**^P < 0.01, respectively; two-tailed *t*-test).

To understand the roles of LTs in *S. oneidensis*, strains lacking each gene were constructed individually. Firstly, we measured growth of each mutant in LB broth medium (Figure 2). Deletion of the *mltB, mltD, mltD2*, and *slt2* genes had little impact on cell growth. In contrast, the other three mutants (the Δ*sltY*, Δ*mltF*, and Δ*mltB2* strains) displayed obviously defects when grown in LB broth. Compared to the wild type, the generation time of the three mutants increased about 20 percent. Genetic complementation was then carried out with the multicopy plasmid pHG101 harboring a copy of *sltY, mltF*, or *mltB2* under the control of their native promoters (Wu et al., 2011). The growth defect of the three mutants was fully recovered by expression of the corresponding genes *in trans* (Figure 2), confirming that growth defects observed in the three mutants were due to the mutation *per se*.

To assess whether growth defects are involved in impaired cell wall integrity, we observed cell shape and determined the susceptibility to hyposmolality. *S. oneidensis* is a typical rod-shaped bacterium and can grow normally in LB minus NaCl. Both cell shape and susceptibility to hyposmolality in all tested mutants were similar to those in the wild type (Figure S2). These results suggest that loss of single LT does not affect the cell wall integrity, growth defects of the Δ*sltY*, Δ*mltF*, and Δ*mltB2* strains are result from other unknown reasons.

### Deletion of *sltY, mltB*, and *mltB2* increases β-lactam resistance

**Figure 3:**
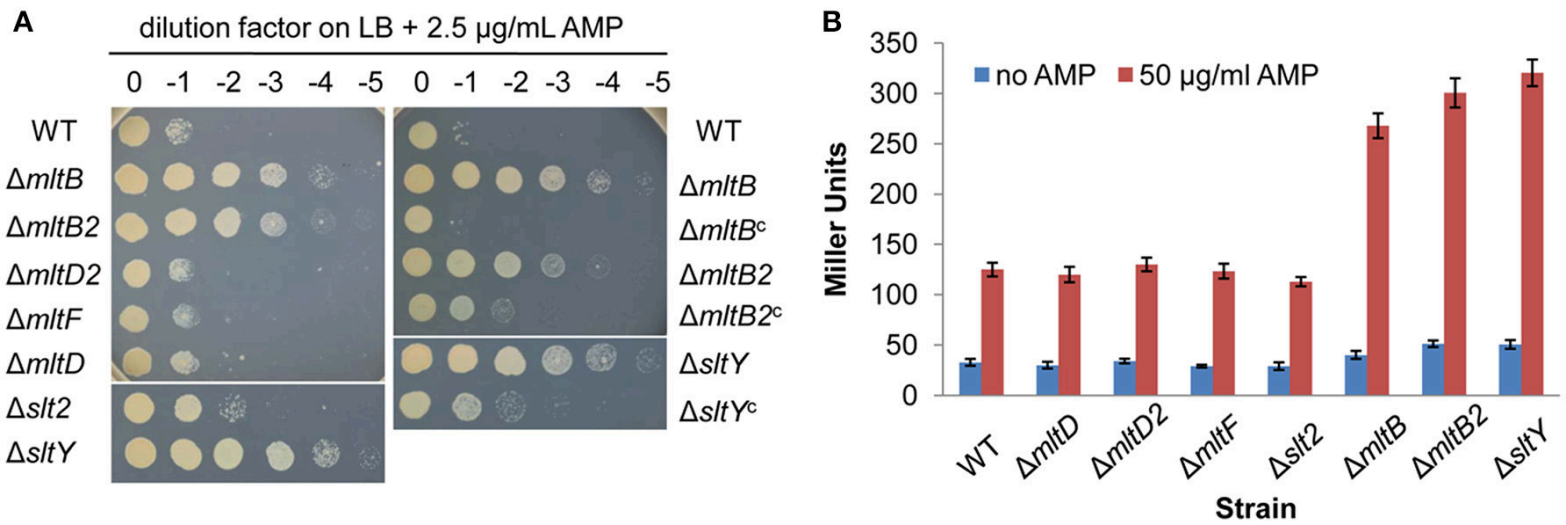
Inactivation of MltB, MltB2 and SltY increased the resistance to AMP. **(A)** AMP susceptibility assay for *S. oneidensis* strains lacking LTs. Cells of late-logarithmic phase (OD600 ≈ 0.6) were used to prepare a decimal dilution series. Three μL of each dilution was spotted onto LB plates supplemented with 2.5 μg/mL AMP. Δ*mltB*^c^, Δ*mltB2*^c^, and Δ*sltY*^c^ represent Δ*mltB*, Δ*mltB2*, and Δ*sltY* that were complemented with pHG101 *in trans*, respectively. **(B)** Improved AMP resistance in Δ*mltB*, Δ*mltB2*, and Δ*sltY* strains is dependent on the expression of *blaA*. Promoter activity of the *blaA* gene (P_*blaA*_) was determined by measuring β-galactosidase (in Miller Units) using the P_*blaA*_*-lacZ* reporter system in the wild type and single LT gene deletion strains. Results are averages for at least three replicates, and the error bars represent standard derivation (SD).

Our previous study showed that major peptidoglycan recycling enzymes are involved in β-lactamase BlaA expression and β-lactam resistance in *S. oneidensis* (Yin et al., 2014). To study whether LTs are also play important roles in this process, the resistance to β-lactams in these LTs mutants were determined. Deletion of the *mltF, mltD, mltD2* and *slt2* genes did not affect the susceptibility to AMP. In contrast, the Δ*sltY*, Δ*mltB2*, and Δ*mltB* strains displayed significantly improved resistance to AMP (Figure 3A). Compared to the wild type, the Δ*sltY*, Δ*mltB2*, and Δ*mltB* strains had 8-, 8-, and 4-fold increases in resistance to AMP, respectively. Besides, the Δ*sltY* and Δ*mltB2* strains had 2-fold increases in resistance to another two tested β-lactams, ceftotaxime (CTX) and imipenem (IMP) (Table 3).

**Table 3:** MICs of β-lactams and specific activities of BlaA in *S. oneidensis* wild type and its derivate strains.

**Strains^a^**	**MIC (μg/mL)^b^**			**β-lactamase activity^c^ ± SD**	
	**AMP**	**CTX**	**IMP**	**No inducer**	**Inducer**
WT	4	0.02	0.5	9 ± 0.5	111 ± 11
Δ*sltY*	32	0.04	1	19 ± 1	140 ± 5
Δ*mltD*	4	0.02	0.5	8 ± 2	104 ± 7
Δ*mltD2*	4	0.02	0.5	10 ± 1	112 ± 4
Δ*mltF*	4	0.02	0.5	6 ± 0.4	108 ±10
Δ*mltB*	16	0.02	0.5	19 ± 0.8	140 ± 4
Δ*mltB2*	32	0.04	1	20 ± 3.5	179 ± 10
Δ*slt2*	4	0.02	0.5	9 ± 0.8	98 ± 3
Δ*sltY*/pHG101	32	0.04	1	–^d^	–
Δ*mltB*/pHG101	16	0.02	0.5	–	–
Δ*mltB2*/pHG101	32	0.04	1	–	–
Δ*sltY*^c^	4	0.02	0.5	7 ± 1.4	89 ± 8
Δ*mltB*^c^	4	0.02	0.5	8 ± 1.1	96 ± 6
Δ*mltB2*^c^	4	0.02	0.5	7 ± 1	90 ± 11
Δ*blaA*	<0.5	<0.005	<0.06	1 ± 0.4	0
Δ*sltY*Δ*blaA*	<0.5	<0.005	<0.06	1 ± 0.5	0
Δ*mltB*Δ*blaA*	<0.5	<0.005	<0.06	2 ± 0.6	0
Δ*mltB2*Δ*blaA*	<0.5	<0.005	<0.06	1 ± 0.5	0
Δ*mltB*Δ*mltB2*	64	0.08	2	45 ± 4	182 ± 7
Δ*sltY*Δ*mltB2*	64	0.08	2	32 ± 1.8	198 ± 7
Δ*ampG*	128	0.08	2	29 ± 4	238 ± 5.1
Δ*mltB*Δ*mltB2*Δ*ampG*	128	0.08	2	98 ± 12	208 ± 4
Δ*mltB2*Δ*sltY*Δ*ampG*	128	0.08	2	92 ± 2	225 ± 10
Δ*mrcA*	128	0.08	2	228 ± 29	243 ± 9
Δ*mltB*Δ*mltB2*Δ*mrcA*	128	0.08	2	253 ± 20	231 ± 6
Δ*mltB2*Δ*sltY*Δ*mrcA*	128	0.08	2	259 ± 10	258 ± 22

a *ΔsltY^c^, ΔmltB^c^, and ΔmltB2^c^ represent ΔsltY, ΔmltB, and ΔmltB2 that were complemented with pHG101 harboring a copy of the corresponding S. oneidensis genes in trans, respectively*.

b *AMP, ampicillin; CTX, cefotaxime; IMP, imipenem*.

c *Nanomoles of nitrocefin hydrolyzed per minute per milligram of protein. Induction was carried out with 200 μg/mL ampicillin for 2 h*.

d *-, not tested*.

More importantly, expression of the *sltY, mltB2*, and *mltB* genes under the control of their native promoters recovered all β-lactam susceptibility to the level of the wild type, indicating that the phenotypes observed in these mutants was due to the intended mutations (Figure 3A and Table 3). These data, collectively, indicate that certain LTs (SltY, MltB2 and MltB) are important players mediating the β-lactam resistance in *S. oneidensis*.

### Improved β-lactam resistance is dependent on BlaA

Production of β-lactamase BlaA confers *S. oneidensis* with natural resistance to certain β-lactams (Yin et al., 2013). In strains lacking AmpG or NagZ, two major peptidoglycan recycling enzymes, BlaA expression is significantly increased, leading to improved β-lactam resistance (Yin et al., 2014). It is reasonable that LTs-mediated β-lactam resistance is also dependent on BlaA.

To test this possibility, the *lacZ* reporter system was employed to determine the activity of the *blaA* promoter (P_*blaA*_) (Yin et al., 2014, 2015). As shown in the Figure 3B, the expression levels of the β-galactosidase driven by the P_*blaA*_ in the Δ*sltY*, Δ*mltB2* and Δ*mltB* strains were substantially higher than that in the wild type under all tested conditions. To confirm, the BlaA activity in all LTs mutants was measured by the nitrocefin hydrolysis method (Table 3; Yin et al., 2014). Our previously study found that β-lactamase activity was hardly detected in the Δ*blaA* strain under all tested conditions, implicating that nitrocefin assay is specific for BlaA in *S. oneidensis*. As expected, deletion of the *mltF, mltD, mltD2*, and *slt2* genes did not affect the BlaA activities under all tested conditions. However, the Δ*sltY*, Δ*mltB2*, and Δ*mltB* strains had higher levels of BlaA activity. Compared to the wild type, the basal levels of BlaA activity of all these three mutants were increased ~2-fold, while the induced levels were increased 1.2~1.6-fold. Notably, expression of the *sltY, mltB2* and *mltB* in the corresponding mutants significantly decreased the BlaA activities under all tested conditions.

In addition, the *blaA* gene was deleted from the Δ*sltY*, Δ*mltB2*, and Δ*mltB* strains. Similar to the Δ*blaA* strain, loss of BlaA completely abolished the β-lactam resistance and BlaA activities of the three LTs mutants (Table 3). Based on these results, we can conclude that the improved β-lactam resistance in the Δ*sltY*, Δ*mltB2*, and Δ*mltB* strains is dependent on BlaA.

### Additive effects of *sltY, mltB*, and *mltB2* on β-lactam resistance

**Figure 4:**
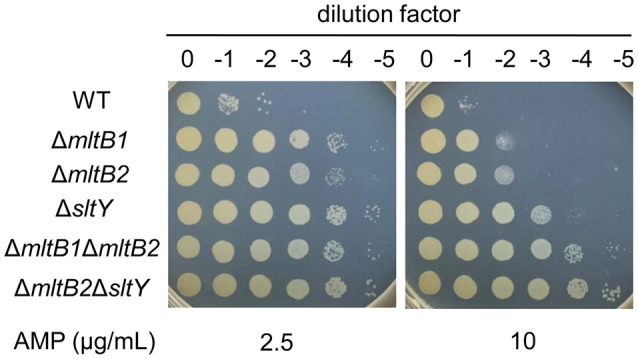
LTs have additive effects on AMP resistance. Cells of late-logarithmic phase (OD600 ≈ 0.6) were used to prepare a decimal dilution series. Three μL of each dilution was spotted onto LB plates supplemented with 2.5 or 10 μg/mL AMP.

Given that different LTs appear to play functionally redundant roles in peptidoglycan degradation, we supposed that LTs may have additive effects on *blaA* expression. To confirm, the Δ*mltB*Δ*mltB2* and Δ*sltY*Δ*mltB2* strains were constructed and subjected to β-lactam susceptibility testing. As shown in Figure 4 and Table 3, both two strains had increased β-lactam resistance compared to the single mutants (Δ*mltB*, Δ*mltB2*, and Δ*sltY*). When compared to the Δ*mltB* and Δ*mltB2* strains, the MICs of the Δ*mltB*Δ*mltB2* strain for all three β-lactams (AMP, CTX, and IMP) were increased 4- and 2-fold, respectively. The Δ*sltY*Δ*mltB2* strain had 2-fold increases in resistance to these β-lactams compared to the single mutants.

Consistently, both the basal and induced levels of BlaA activity were increased in the two double mutants. The Δ*mltB*Δ*mltB2* and Δ*sltY*Δ*mltB2* strains had basal levels of BlaA activity ~2.2- and ~1.7-fold higher than either of its single-mutation parent strains, respectively. By induction, the BlaA activity of the Δ*mltB*Δ*mltB2* strain was 1.3-fold higher than that of the Δ*mltB* strain. Similarly, the BlaA activity of the Δ*sltY*Δ*mltB2* strain was ~1.4-fold higher than that of the Δ*sltY* strain. These results suggest that SltY, MltB and MltB2 have additive effect on *blaA* expression and β-lactam resistance.

### Loss of AmpG enhances LTs-mediated BlaA activity at basal levels

In *S. oneidensis*, loss of AmpG increases the basal level of *blaA* expression, implying that intermediate products transported across AmpG permease are likely to be the repressors rather than inducers for *blaA* expression (Yin et al., 2014). To evaluate the effects of AmpG on LTs-mediated *blaA* expression, the Δ*mltB*Δ*mltB2*Δ*ampG* and Δ*sltY*Δ*mltB2*Δ*ampG* strains were constructed for antibiotic susceptibility testing (Table 3). The two strains, together with the Δ*ampG* strain, displayed similar levels of resistance to all tested β-lactams, which had 2-fold increases in resistance to AMP when compared to the strains lacking two LTs (Δ*mltB*Δ*mltB2* and Δ*sltY*Δ*mltB2*). Notably, deletion of *ampG* from the two double mutants increased the basal levels of BlaA activity. Therefore, loss of AmpG further increases the basal levels of *blaA* expression and β-lactam resistance in strains lacking LTs.

### PBP1a-mediated induction of *blaA* expression is independent of the LTs

Our previous study demonstrated that deletion of *mrcA* (encoding PBP1a) drastically enhances *blaA* expression in *S. oneidensis* even in the absence of β-lactams, which is independent of AmpG (Yin et al., 2015). Although significant, the increase in β-lactamase activity after inactivating LTs is much lower than that produced by PBP1a inactivation. To explore the involvement of LTs in PBP1a-mediated *blaA* expression, we deleted the *mrcA* gene from both Δ*mltB*Δ*mltB2* and Δ*sltY*Δ*mltB2* strains and performed the antibiotic susceptibility testing (Table 3). The resistance to β-lactams of both the Δ*mltB*Δ*mltB2*Δ*mrcA* and Δ*sltY*Δ*mltB2*Δ*mrcA* strains was identical to those of the Δ*mrcA* strain. Compared to the strains lacking two LTs, both the two triple mutants had 2-fold increases in resistance to AMP. Consistently, deletion of *mrcA* significantly increased the basal levels of BlaA activity of the strains lacking two LTs. Both the basal and induced levels of BlaA activity of the triple mutants were comparable to those of the Δ*mrcA* strain (Table 3). These results suggest that PBP1a-mediated *blaA* expression is independent of LTs. In other words, PBP1a functionally overrides LTs in *blaA* expression.

## Discussion

The regulatory mechanism for *blaA* induction in *S. oneidensis* is distinct from the *ampR*-*ampC* paradigm. Nonetheless, our previous studies demonstrated that the expression of *blaA* is also related to peptidoglycan maintenance, turnover and recycling (Yin et al., 2013, 2014, 2015). In contrast to the *ampR*-*ampC* paradigm, inactivation of major peptidoglycan recycling enzymes, AmpG and NagZ, improved the *blaA* expression and subsequent β-lactam resistance. Here we evaluated whether LTs are involved in β-lactam resistance in *S. oneidensis*. This bacterium harbors seven putative LTs. All these LTs are effectively expressed in the wild type background. Our results demonstrated that only family 1 LT, SltY and family 3 LTs, MltB and MltB2, are related to the *blaA* expression. Loss of these LTs improves the BlaA activities under basal and induced conditions, resulting in increased β-lactam resistances.

*S. oneidensis* LTs also play different roles in bacterial cell growth. Strains lacking SltY, MltB2 or MltF displayed a certain growth defect, while other LTs have no effects on growth. Our results suggest that *S. oneidensis* LTs have different physiological functions, although they appear to be redundant in cleavage of glycan strains. Notably, MltB and MltB2 are both family 3 LTs, their roles in cell growth are also different. Consistently, the distinct roles of LTs have been observed in other bacteria. For example, *Salmonella enterica* serovar Typhimurium contains seven putative family 1 LTs, whereas only two of them, MltE and MltC, are specifically involved in the regulation of biofilm formation (Monteiro et al., 2011). At least three reasons might contribute to the distinct roles of LTs. Firstly, the expression of LTs may be changed by different growth phase and physiological conditions. Secondly, two forms of LTs (soluble and membrane-bound) may carry out either exolytic or endolytic reaction. In addition, it is possible that LTs have different substrates preferences, which have been observed in *E. coli* (Lee et al., 2013).

The effects of LTs on β-lactam resistance have been evaluated in some other *ampR*-*ampC* harboring Gram-negative bacteria. In *P. aeruginosa*, loss of family 3 LTs SltB1 and MltB increases β-lactam resistance, while loss of Slt decreases β-lactam resistance. However, inactivation of these LTs did not affect the uninduced and induced AmpC activities (Cavallari et al., 2013; Lamers et al., 2015). On the contray, deletion of *mltD1* increased the uninduced β-lactamase activity in *Stenotrophomonas maltophilia*, which results from *mltB1* and *mltD2* upexpression and depends on the *ampG*-*nagZ*-*ampR* regulatory circuit (Huang et al., 2015; Wu et al., 2016). Notably, inactivation of LTs in these bacteria compromise cell envelope integrity, results in susceptibility to β-lactams and other antibiotics (Lamers et al., 2015; Wu et al., 2016).

**Figure 5:**
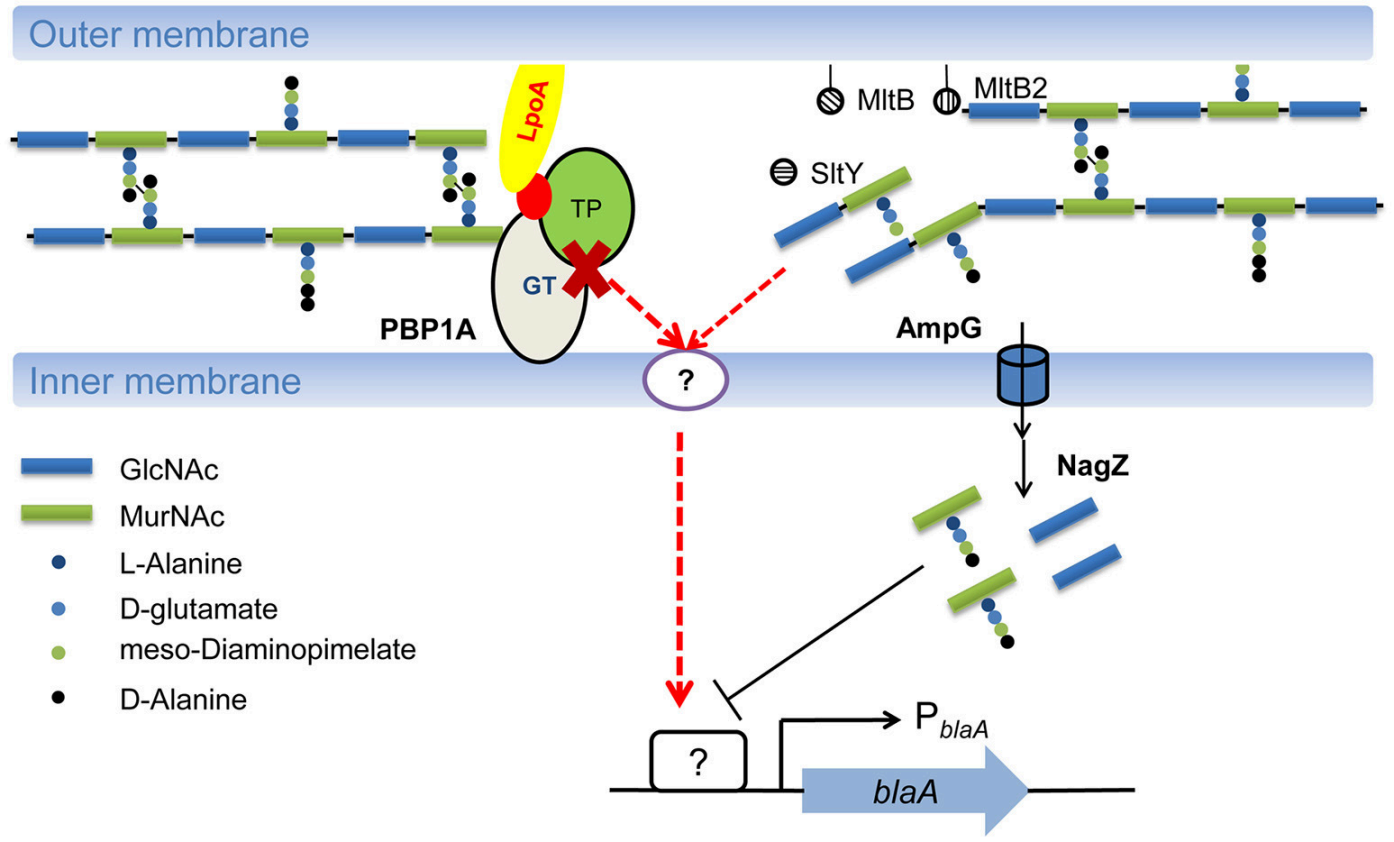
Model of regulatory mechanism for *blaA* induction in *S. oneidensis*. *S. oneidensis* possesses two pathways for *blaA* regulation. One is an AmpG-NagZ-dependent repressible pathway, which is also LT dependent. Another one is AmpG-NagZ-independent inducible pathway, which is dependent on PBP1a. PBP1a is responsive to the presence of antibiotics in the periplasm, impaired peptidoglycan production by PBP1a compromises cell envelope integrity, and then activates an unknown TCS, eventually results in expression of *blaA* in *S. oneidensis*. Moreover, it cannot be excluded that LTs inactivation disturbs peptidoglycan homeostasis, which is then monitored an unknown TCS to induce the expression of *blaA*.

Our studies on *S. oneidensis* showed that deletion of three LTs (MltB, MltB2 and SltY) increase β-lactam resistance. However, the underlying mechanism is quite different from that in *P. aeruginosa* or *S. maltophilia*. The upregulation of *blaA* contributes to LTs-mediated β-lactam resistance in *S. oneidensis*. The regulatory mechanism for *blaA* induction is unique because of the lack of an AmpR homolog (Yin et al., 2014). More importantly, *S. oneidensis* contains an AmpG-NagZ-dependent repressible pathway and AmpG-independent inducible pathway for *blaA* expression, since loss of AmpG increases the basal level of BlaA activity and remains inducible (Figure 5; Yin et al., 2014). Peptidoglycan degradation fragments transported by AmpG might function as repressors (rather than inducers for the *ampR*-*ampC* paradigm) for *blaA* induction. LTs are responsible for the cleavage of glycan strands and production of GlcNAc-1,6-anhMurNAc peptides. It is reasonable that the repressors for *blaA* induction are derived from these LTs. Although the AmpR homolog is absent in *S. oneidensis*, genome annotation demonstrated that this bacterium possesses at least 22 putative LysR-type transcriptional regulators. Unlike AmpR, certain regulators could bind to these repressors, thus inhibiting β-lactam expression in the absence of inducer. In general, the AmpG-NagZ-dependent repressible pathway for *blaA* expression is also LTs dependent.

Given that inactivation of *mrcA* (encoding PBP1a) derepresses the expression of *blaA* in the strain lacking AmpG, the AmpG-independent inducible pathway for *blaA* expression may be dependent on PBP1a (Yin et al., 2015). In this study, we found that PBP1a also functionally overrides LTs in *blaA* expression, implicating that PBP1a-depedent inducible pathway is LTs independent. As the primary target for β-lactams, PBP1a is responsive to the presence of antibiotics in the periplasm. In *P. aeruginosa*, inactivation of a nonessential PBP triggers the activation of CreBC two-component system (TCS), which in turn play an important role in β-lactam resistance (Zamorano et al., 2014). In parallel, BlrAB, a homolog of CreBC, are involved in β-lactamase expression and β-lactam resistance in *Aeromonas* spp. (Niumsup et al., 2003). Recently, TCSs that sense β-lactams (VbrKR) or cell wall damage (WigKR) have been characterized in *Vibrio*, which are responsible for β-lactam resistance or tolerance (Dörr et al., 2016; Li et al., 2016). However, the VbrKR and CreBC/BlrAB homologs are absent in *S. oneidensis*. It is notable that *S. oneidensis* possesses a large number of TCSs, including Cpx and Rcs (Laubacher and Ades, 2008; Weatherspoon-Griffin et al., 2010), two systems that monitor cell envelope stress. It is likely that impaired peptidoglycan production by PBP1a compromises cell envelope integrity, and then activates an unknown TCS, eventually results in expression of *blaA* in *S. oneidensis* (Figure 5).

A recent study showed that inactivation of LTs alters the outer membrane permeability in *S. maltophilia* (Wu et al., 2016). Although our results suggested that deletion of LTs in *S. oneidensis* did not affect cell wall integrity, it cannot be excluded that LT inactivation disturbs peptidoglycan homeostasis, which is then monitored an unknown TCS to induce the expression of *blaA* (Figure 5).

It has been proposed that the combination of LT inhibitors and β-lactams can be used to treat *ampR*-*ampC* harboring pathogens (Kraft et al., 1999). However, the LTs in different bacteria may play different roles in β-lactam resistance. Especially, loss of three LTs in *S. oneidensis* increased the expression of class D β-lactamase BlaA, which is regarded as progenitor of carbapenem-hydrolyzing oxacillinase in clinically relevant Gram-negative pathogens (Poirel et al., 2004). Therefore, it is importance to understand the roles of LTs in β-lactam resistance in bacteria that are phylogenetically more diverse. In addition, the species specific effects of LT inhibitors should be carefully considered.

## Author contributions

JY, ZY, JQ, and HG analyzed data, conceived the idea and designed the project. JY, YYS, and YJS carried out the experiments. JY and HG wrote the paper.

### Conflict of interest statement

The authors declare that the research was conducted in the absence of any commercial or financial relationships that could be construed as a potential conflict of interest.
