# Supplementary figures and images for: Deletion of Lytic Transglycosylases Increases Beta-Lactam Resistance in Shewanella oneidensis
Source: Front Microbiol. 2018 Jan 22;9:13. doi: 10.3389/fmicb.2018.00013 (PMC5786531; doi:10.3389/fmicb.2018.00013)

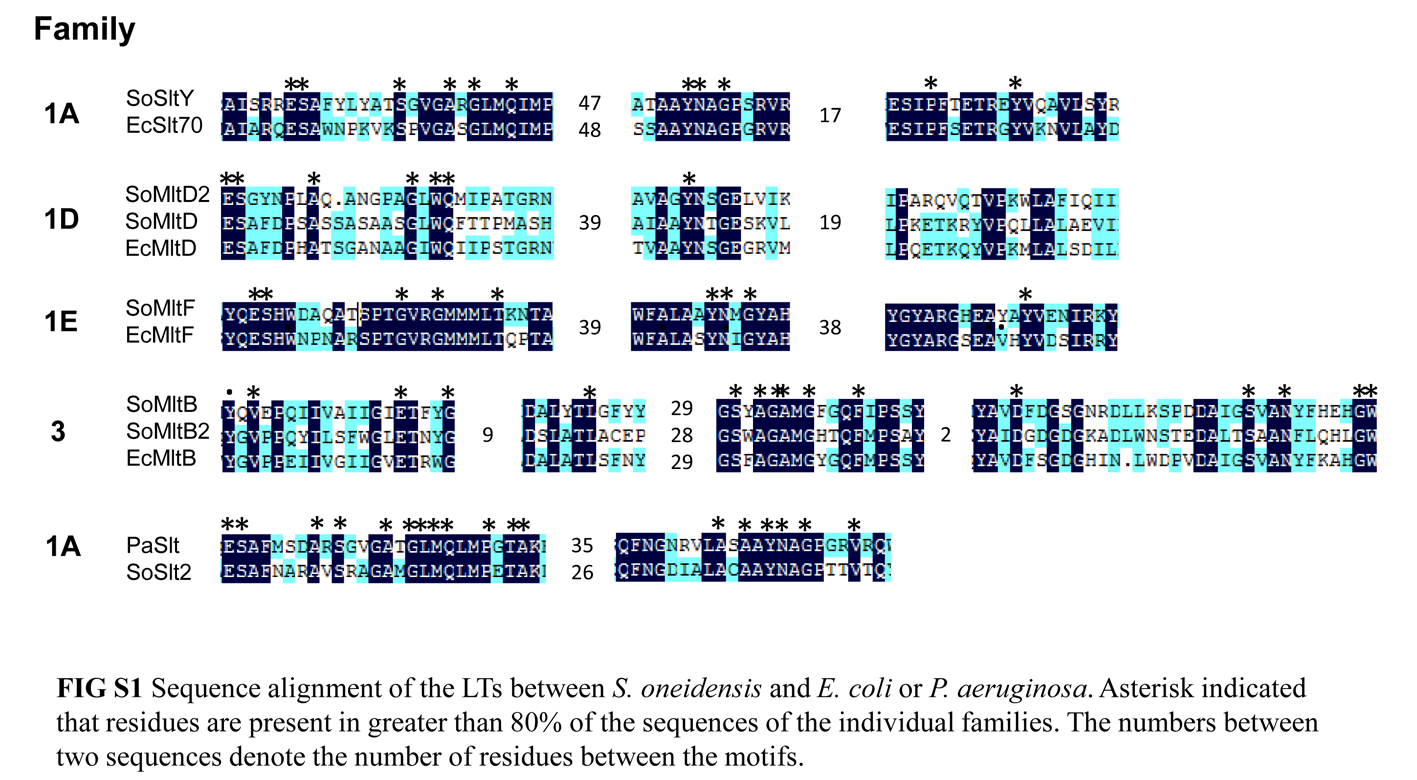

Supplement: Supplementary file 1 [file Image1.TIF]

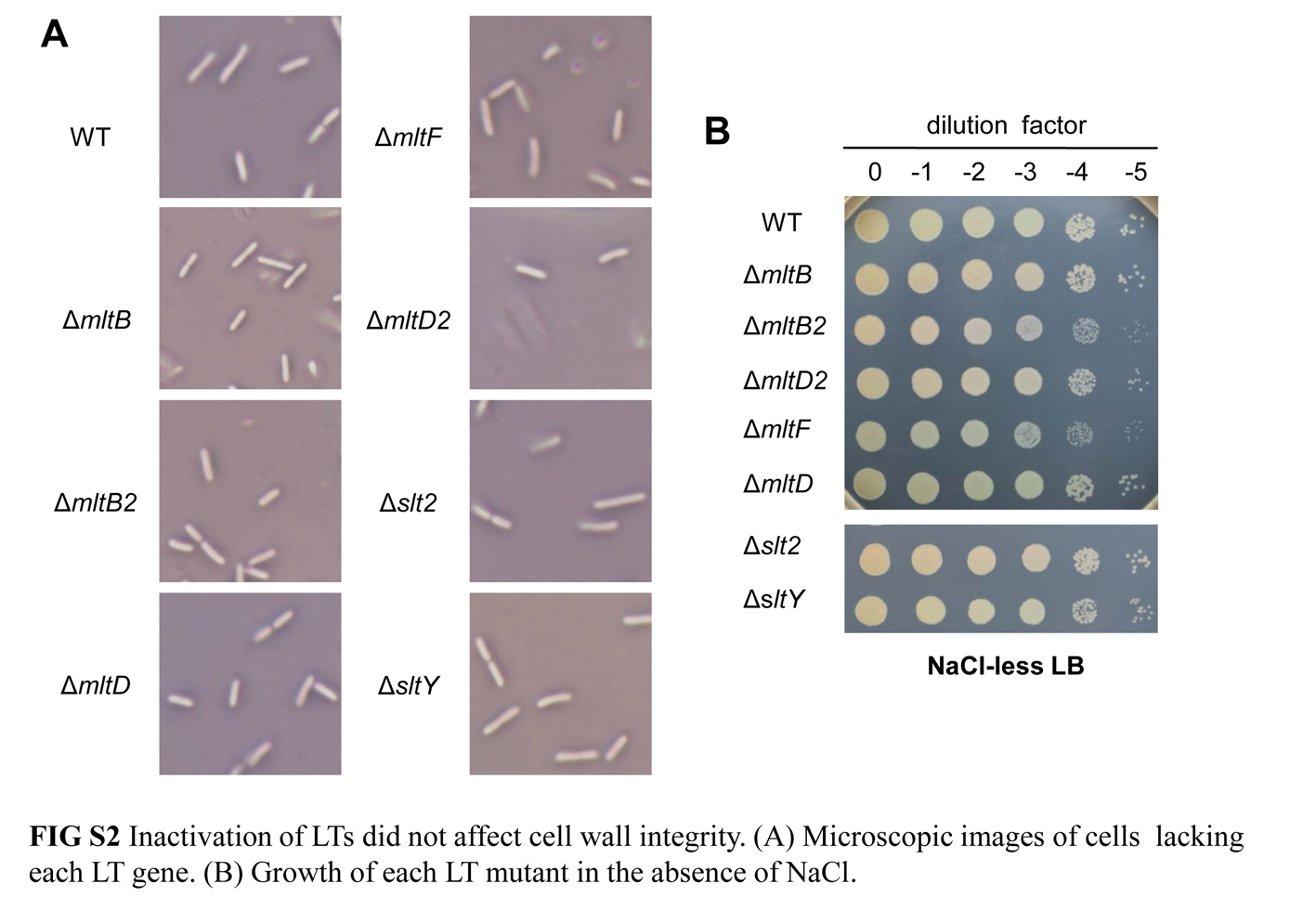

Supplement: Supplementary file 2 [file Image2.TIF]
