# Supplementary material for: Deletion of Lytic Transglycosylases Increases Beta-Lactam Resistance in Shewanella oneidensis
Source: Front Microbiol. 2018 Jan 22;9:13. doi: 10.3389/fmicb.2018.00013 (PMC5786531; doi:10.3389/fmicb.2018.00013)
Supplement: Supplementary file 3 [file Table1.DOCX]

**Supplemental materials**

**Inactivation of Lytic transglycosylases increase beta-lactam resistance in *Shewanella oneidensis***

Jianhua Yin^1,2,3^*, Yiyang Sun^2^, Yijuan Sun^2^, Zhiliang Yu^1^, Juanping Qiu^1^, Haichun Gao^2^*

^1^College of Biotechnology and Bioengineering, Zhejiang University of Technology, Hangzhou, China

^2^Institute of Microbiology and College of Life Sciences, Zhejiang University, Hangzhou, China

^3^College of Life Sciences, Nanchang University, Nanchang, China

**Table S1 All primers used in this study**

| Mutagenesis |  |
| --- | --- |
| Δ*mltB*-5F | GGGGACAAGTTTGTACAAAAAAGCAGGCTGCGGTCTTTGGGGTAAAGGA |
| Δ*mltB*-5R | ATGCACGACTTCTGAGCAATAGCACTGCCAATGGAGCTAA |
| Δ*mltB*-3F | ATTGCTCAGAAGTCGTGCATTCACCCGTTACAATCGTAGTCC |
| Δ*mltB*-3R | GGGGACCACTTTGTACAAGAAAGCTGGGTGGACGTCTCATAGCCGTGAA |
| Δ*mltB2*-5F | GGGGACAAGTTTGTACAAAAAAGCAGGCTGATGAGCATTGTGTTGGCGG |
| Δ*mltB2*-5R | TGTCGAGCTGCGAGATCACTTTGGCGAGCTAGAACAAGCA |
| Δ*mltB2*-3F | AGTGATCTCGCAGCTCGACATCCGGCTTTGATGTCGGTAA |
| Δ*mltB2*-3R | GGGGACCACTTTGTACAAGAAAGCTGGGTCCGCGATGAGATACGTCCAG |
| Δ*mltD*-5F | GGGGACAAGTTTGTACAAAAAAGCAGGCTCGCTAACGTCTGCACTCTCT |
| Δ*mltD*-5R | CAATGACGAGTTGAGGCAATGCTAATGCATGGGGTTGACG |
| Δ*mltD*-3F | ATTGCCTCAACTCGTCATTGAGAGCATCATCAAGCCAGGTC |
| Δ*mltD*-3R | GGGGACCACTTTGTACAAGAAAGCTGGGTGGTGCTGAACAAGGACGATTC |
| Δ*mltD2*-5F | GGGGACAAGTTTGTACAAAAAAGCAGGCTCGCCAACCATAGTGGACAGT |
| Δ*mltD2*-5R | CGAGATAAGGTGCGATCACTTGTCTAACGTCTGGCAACCC |
| Δ*mltD2*-3F | AGTGATCGCACCTTATCTCGACCGTGGCTGAACTCCTAGA |
| Δ*mltD2*-3R | GGGGACCACTTTGTACAAGAAAGCTGGGTCAACAGGCTTGTAACTGACGC |
| Δ*mltF*-5F | GGGGACAAGTTTGTACAAAAAAGCAGGCTTTACGCAGGCCTCCATTAGC |
| Δ*mltF*-5R | TGTCTAGCACGTCCTGCAATTTCCACGGTCTCTTGCTGAC |
| Δ*mltF*-3F | ATTGCAGGACGTGCTAGACATAGCGAAGAAGTTGCACCGA |
| Δ*mltF*-3R | GGGGACCACTTTGTACAAGAAAGCTGGGTCGGCGAGATGCGTTGTTATT |
| Δ*sltY*-5F | GGGGACAAGTTTGTACAAAAAAGCAGGCTTGCCACGTTAAGATTTGCTGT |
| Δ*sltY*-5R | AGAGCCTCCTCATTTCGTATGAAACCCGAGAATATGTCCAA |
| Δ*sltY*-3F | ATACGAAATGAGGAGGCTCTAACGCAGTCTCTGATACATAGGTG |
| Δ*sltY*-3R | GGGGACCACTTTGTACAAGAAAGCTGGGTCGAGAAAACCTATCACAGTGCG |
| Δ*slt2*-5F | GGGGACAAGTTTGTACAAAAAAGCAGGCTATCGCGGTGGTTTAGCCAAT |
| Δ*slt2*-5R | AGGCTCGCATTTGGATCAATCAGCAAACACGGTTGCAAGA |
| Δ*slt2*-3F | ATTGATCCAAATGCGAGCCTAAGCGTTACCGTGACACCAA |
| Δ*slt2*-3R | GGGGACCACTTTGTACAAGAAAGCTGGGTATCAATCCACCGTGGCAACT |
| Complementation |  |
| pHG101-SltY-F | CGAATTCTGCTAAACAAGCAGGTCGGA |
| pHG101-SltY-R | CGGATCCTGTGTGCGTTTGCCGATTTT |
| pHG101-MltB-F | CGAATTCCAGGCGGTCTTTGGGGTAAA |
| pHG101-MltB-R | CGGATCCGCAACTGAGCATCAAGAGCG |
| pHG101-MltB2-F | CGAATTCCCCATTATACGGCAACGGCT |
| pHG101-MltB2-R | CCCTCGAGCTGCGGGCGATTGCATAAAA |
| pHG101-MltF-F | CGGATCCATGACTCGATTTTTGTTCGC |
| pHG101-MltF-R | CCCAAGCTTTGCGGCCGTTTGGGGGACTA |
| qRT-PCR |  |
| blaA-qF | ACTCGGTCGTGCCCGTGTATCA |
| blaA-qR | ATCCGAATGCCGCCATCGAGC |
| Promoter activity |  |
| SltY-5P | CGAATTCCACCATCAATAATATTTGGCG |
| SltY-3P | CGGATCCGTAATCATGGTCATGCAGAATTTGCCTCCGGGCA |
| Slt2-5P | CGAATTCATCGCGGTGGTTTAGCCAAT |
| Slt2-3P | CGGATCCGTAATCATGGTCATCAAAGTCCCGTGTATTCGTT |
| MltB1-5P | CGAATTCCAGGCGGTCTTTGGGGTAAA |
| MltB1-3P | CGGATCCGTAATCATGGTCATTAAAAAGTCCCTAAAAT |
| MltB2-5P | CGAATTCCCCATTATACGGCAACGGCT |
| MltB2-3P | CGGATCCGTAATCATGGTCATCAAATCAATTACTCCTCAAT |
| MltD1-5P | CGAATTCAACGGGTAATCCCTTGTAAT |
| MltD1-3P | CCCAAGCTTGTAATCATGGTCATGATTGTCCTGTAATTTTTGA |
| MltD2-5P | CGAATTCGATTGGAGCGATCAATTAAT |
| MltD2-3P | CGGATCCGTAATCATGGTCATGGAAAACGATAGTCCTCTGT |
| MltF-5P | CGAATTCTAAATGAACATACTCAGCAT |
| MltF-3P | CGGATCCGTAATCATGGTCATTAAATACACTTTATTAAATA |
